# Non-classical polyinterhalides of chlorine monofluoride: experimental and theoretical characterization of [F(ClF)_3_]^−^†

**DOI:** 10.1039/d1cc01088c

**Published:** 2021-04-07

**Authors:** Patrick Pröhm, Nico Schwarze, Carsten Müller, Simon Steinhauer, Helmut Beckers, Susanne M. Rupf, Sebastian Riedel

**Affiliations:** Freie Universität Berlin, Department for Chemistry and Biochemistry Fabeckstr. 34/36 Berlin Germany s.riedel@fu-berlin.de

## Abstract

We present the synthesis and characterization of the first non-classical Cl(i) polyinterhalide [NMe_4_][F(ClF)_3_] as well as the homologous polychloride [NPr_3_Me][Cl_7_]. Both salts were obtained from the reaction of the corresponding ammonium chlorides with ClF or Cl_2_, respectively. Quantum-chemical investigations predict an unexpected planar structure for the [F(ClF)_3_]^−^ anion.

## Abstract 2

The [F(ClF)_3_]^−^ anion was synthesized from an organic chloride salt and chlorine monofluoride. Comparison between the [F(ClF)_3_]^−^ anion and other permutated chlorine fluorine heptahalides reveals the unique effect of a central fluoride ion.

## Graphical abstract

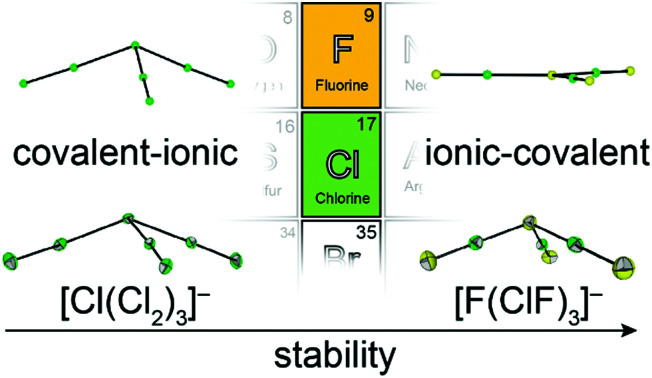

Fluoridopolyhalogen chemistry is experiencing a renaissance. In the last few decades, several binary fluoridopolyhalogenates were structurally described. They can be divided into two conceptually different groups, classical and non-classical interhalides.^1^ Classical interhalides have a central, more electropositive halogen atom surrounded by more electronegative halogens. The majority of the known fluoridohalides belong to this group, such as [IF_2_]^−^,^2,3^ [IF_4_]^−^,^3,4^ [IF_6_]^−^,^5^ [IF_8_]^−^,^6^ [BrF_2_]^−^,^7,8^ [BrF_4_]^−^,^8–11^ [BrF_6_]^−^,^10,11^ [ClF_2_]^−^,^12^ [ClF_4_]^−^,^11,13,14^ and [ClF_6_]^−^.^14^ The valence shell pair repulsion (VSEPR) model can well predict the linear structures of the [XF_2_]^−^ anions and square planar shape of [XF_4_]^−^, but not the undistorted octahedral structures of the [XF_6_]^−^ (X = Br, Cl) anions.^10^ Non-classical interhalides have more electronegative central halogen atoms surrounded by more electropositive (poly)halogen ligands. The known fluoridohalides of this group are [F(IF_5_)_3_]^−^,^10^ [F(BrF_3_)_2_]^−^,^15,16^ and [F(BrF_3_)_3_]^−^.^16^ The Kraus group recently synthesized the first non-classical fluoridochlorate(iii), the [F(ClF_3_)_3_]^−^ anion obtained by solvolysis of CsF in ClF_3_.^17^1
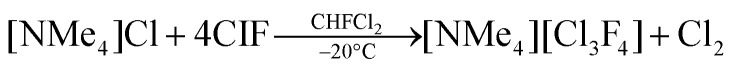
Here, we present an unprecedented Cl(I) compound, [NMe_4_][F(ClF)_3_]. With a formal [F(ClF)_3_]^−^ anion, it belongs to the non-classical interhalides. It was synthesized *via* the exposure of tetramethylammonium chloride to an excess of chlorine monofluoride in dichlorofluoromethane at low temperatures (eqn (1)). Initially, ClF likely oxidizes the chloride anion to yield elemental chlorine and a fluoride anion, which is eventually coordinated by three ClF molecules.

We were able to grow single crystals suitable for X-ray diffraction at −80 °C. [NMe_4_][F(ClF)_3_] crystallized in the orthorhombic space group *Pna*2_1_, as shown in Fig. 1. The anion consists of a central fluoride anion F1 coordinated by three ClF molecules in a pyramidal shape. Two of the bond lengths to F1 are almost identical (*d*(F1-Cl2) = 219.4(2) pm, *d*(F1–Cl3) = 219.5(1) pm), whereas the bond to the third ClF ligand is approximately 6 pm shorter (*d*(F1–Cl1) = 213.9(2) pm). The inverse trend is observed for the Cl–F bond lengths of the ligands: *d*(Cl1–F2) = 169.9(2) pm, *d*(Cl2–F3) = *d*(Cl3–F4) = 168.1(1) pm. The Cl–F bond length of neat ClF in the solid-state is 162.8(1) pm.^18^ The elongation of the di- or interhalogen ligand bond is well understood in polyhalide chemistry. It can be attributed to the interaction between the lone pairs of the central fluoride anion with the σ*(Cl–F) orbital in the ligand.^1^ Donation of electron density into this antibonding orbital weakens the corresponding bond. Hence, stronger halide-ligand-interactions result in a more pronounced weakening of the ligand bond. The Cl–F1–Cl bond angles are in the range of 103.87(6)° and 108.86(6)°, and the Cl1–F1–Cl2–Cl3 dihedral angle is 112.12(9)°. The counterion [NMe_4_]^+^ forms three short hydrogen bonds to F1 (see Fig. 1 and Fig. S4 for the Hirshfeld surface, ESI†). Overall, the three hydrogen bonds, together with the three ClF ligands, result in a distorted octahedral coordination sphere for the central F1 anion (Fig. S3, ESI†).

**Fig. 1:**
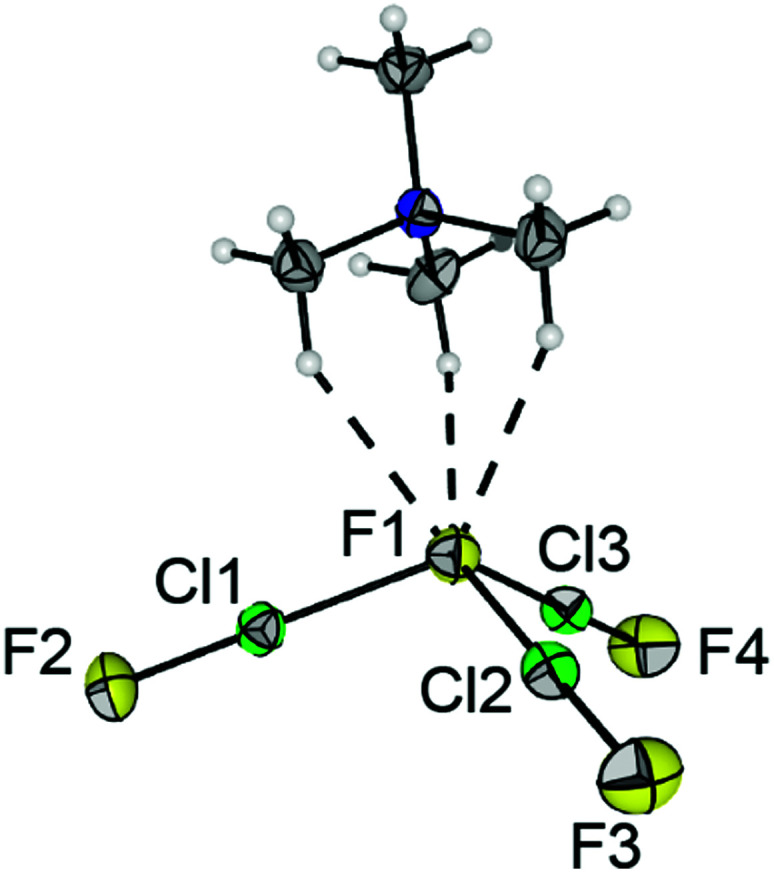
Section of the solid-state structure of [NMe_4_][F(ClF)_3_]. Displacement ellipsoids are shown at the 50% probability level. Color code: yellow = fluorine, green = chlorine, blue = nitrogen, grey = carbon, white = hydrogen. Selected bond lengths [pm] and angles [°]: F1–Cl1 213.9(2), F1–Cl2 219.4(2), F1–Cl3 219.5(1), Cl1–F2 169.9(2) Cl2–F3 168.1(1), Cl3–F4 168.1(1), F1⋯H in the range of 248 to 250, dihedral angle Cl1–F1–Cl2–Cl3 112.12(9).

The Raman spectrum of crystalline [NMe_4_][F(ClF)_3_] (Fig. 2, bottom, full spectrum see Fig. S1, ESI†) shows three bands at 675, 641, and 615 cm^−1^, which are attributed to the stretching vibrations of the Cl–F ligands. The vibrational band of gaseous ClF is reported at 772 cm^−1^.^19^ The red shift is expected due to the weakened inter-ligand bond, consistent with the structural parameters mentioned above. This assignment is supported by periodic solid-state calculations using the CRYSTAL17^20^ program and the B3LYP DFT functional (Fig. 2, for full spectrum see Fig. S8 and Table S2, for computational details see the ESI†).2
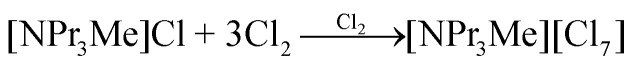


**Fig. 2:**
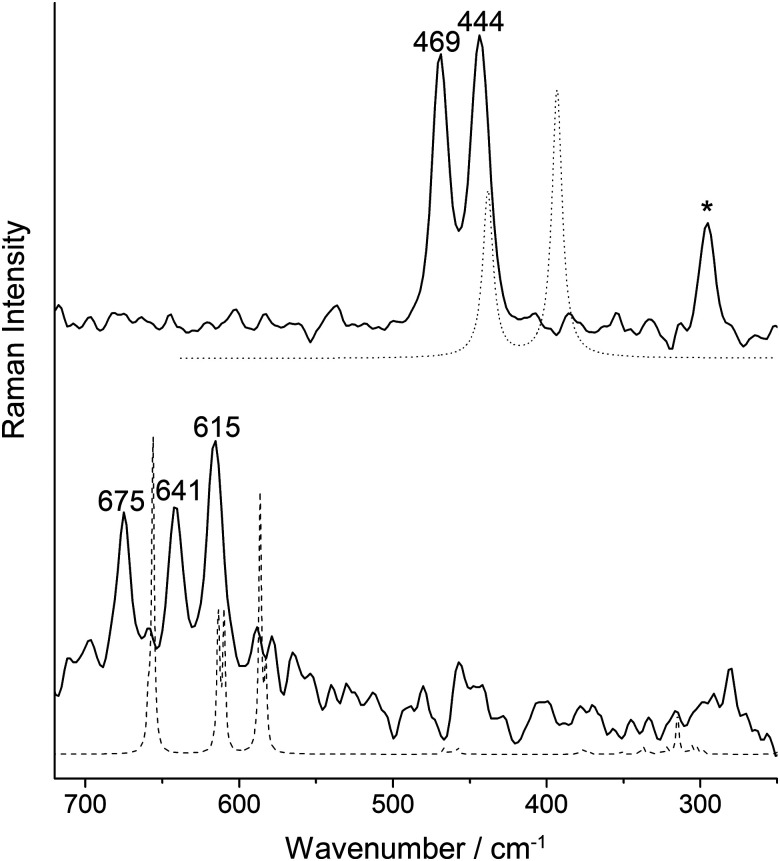
Comparison of the Raman spectra of [NMe_4_][F(ClF)_3_] (bottom, solid line) and [NPr_3_Me][Cl_7_] (top, solid line) with their computed spectra obtained at the B3LYP level (dashed line: solid-state calculation, dotted line: gas-phase calculation). The decomposition product [Cl_3_]^−^ is marked by an asterisk.

The analogous treatment of [NPr_3_Me]Cl with elemental chlorine instead of ClF leads to the polychloride anion formation of [NPr_3_Me][Cl_7_] (eqn (2)). Fig. 3 shows a section of the solid-state structure of [NPr_3_Me][Cl_7_]. It crystallized in the space group *P*1̄. This structure again shows a pyramidal conformation of the anion. Analogously, it can be interpreted as a complex between a central chloride ion with three dichlorine ligands [Cl(Cl_2_)_3_]^−^. The bond lengths between the central chloride ion Cl1 and the chlorine ligands are 276.0(1)–277.4(1) pm. The bond lengths within the Cl_2_ ligands are 202.7(1)–203.8(1) pm, elongated by 4–5 pm compared to solid Cl_2_ (198.4(1) pm).^21^ The anion has two smaller and one significantly larger Cl–Cl1–Cl angles: Cl2–Cl1–Cl6: 82.41(2)°, Cl4–Cl1–Cl2: 93.69(2)°, and Cl4–Cl1–Cl6: 140.25(2)°. The Cl2–Cl1–Cl4–Cl6 dihedral angle is 82.55(4)°. There are five close Cl1⋯H–C contacts to two cations (Fig. 3 and Fig. S5 for the Hirshfeld surface, ESI†). The Raman spectrum of crystalline [NPr_3_Me][Cl_7_] shows two pronounced inter-ligand Cl–Cl stretching vibrations at 469 and 444 cm^−1^, which is consistent with the calculated Raman spectrum for the isolated free [Cl_7_]^−^ anion (Fig. 2, top).

**Fig. 3:**
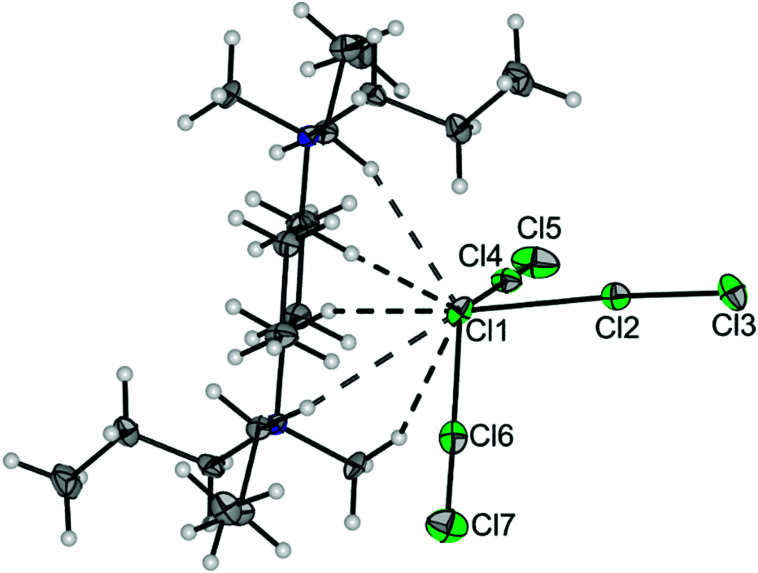
Section of the solid-state structure of [NPr_3_Me][Cl_7_] showing one anion and two cations [one at (*x*, *y*, *z*) and one at (−*x*, 1 − *y*, 1 − *z*)]. Displacement ellipsoids are shown at the 50% probability level. Color code: green = chlorine, blue = nitrogen, grey = carbon, white = hydrogen. Selected bond lengths [pm] and angles [°]: Cl1–Cl2 277.08(8), Cl1–Cl4 275.97(6), Cl1–Cl6 277.36(6), Cl2–Cl3 203.80(8), Cl4–Cl5 203.28(7), Cl6–Cl7 202.66(8), Cl2–Cl1–Cl6 82.41(2), Cl4–Cl1–Cl2 93.69(2), Cl4–Cl1–Cl6 140.25(2), Cl2–Cl1–Cl4–Cl6 82.55(4), Cl⋯H in the range 2.77 to 2.90.

The direct comparison of the [F(ClF)_3_]^−^ and [Cl_7_]^−^ solid-state structures reveals a significantly wider dihedral angle and stronger bonds between the central anion and the ligands in the fluoridochlorate. Elimination of a ClF ligand in [F(ClF)_3_]^−^ is significantly more endothermic than the loss of a Cl_2_ ligand in [Cl_7_]^−^ (Table 1). This is likely due to a higher acidity of ClF and the higher basicity of the central fluoride ion. Calculated halogen-elimination energies for [F(Cl_2_)_3_]^−^ and the hypothetical [Cl(ClF)_3_]^−^ show the same trend (Table 1): ClF elimination is always more endothermic than Cl_2_ elimination and the dihalogen bond to the fluoride anion is stronger than the corresponding bond to Cl^−^.

**Table 1:** Thermochemical data for the decomposition of different heptahalides calculated at different computational levels using the def2-TZVPP basis set at the DFT and MP2 levels, and individual basis sets at the CCSD(T) level (see ESI). Energies are given in kJ mol^−1^. A positive value implies an endothermic reaction

Reaction	B3LYP-D3BJ	SCS-MP2	CCSD(T)
[F(ClF)_3_]^−^ → [F(ClF)_2_]^−^ + ClF	68.5	54.9	63.3
[Cl(Cl_2_)_3_]^−^ → [Cl(Cl_2_)_2_]^−^ + Cl_2_	41.8	32.5^22^	—
[Cl(ClF)_3_]^−^ → [Cl(ClF)_2_]^−^ + ClF	62.6	34.7	53.1
[F(Cl_2_)_3_]^−^ → [F(Cl_2_)_2_]^−^ + Cl_2_	48.8	—	48.7

Unlike the free [Cl(Cl_2_)_3_]^−^ and [Cl(ClF)_3_]^−^ anions and in contrast to the prediction from the VSEPR model the fluoridopolyhalogenates [F(ClF)_3_]^−^ and [F(Cl_2_)_3_]^−^ anions show planar *D*_3h_ molecular structures at the CCSD(T)/aug-cc-pVTZ level, indicating that the pyramidal structure of the title compound in the solid state is most likely due to the formation of hydrogen bonds to the counter ion. Indeed, a relaxed surface scan for [F(ClF)_3_]^−^ revealed a rather flat potential surface with a planar minimum and only a small energy (1.5 kJ mol^−1^) is required for its pyramidalization from a local minimum structure with *C*_3v_ symmetry to the global minimum with *D*_3h_ (Fig. S6, ESI†). Also, the inversion of the [Cl(Cl_2_)_3_]^−^ anion *via* a planar transition state requires a low energy barrier of only 2 kJ mol^−1^ at the MP2/def2-TZVPP level (Fig. S6, ESI†).

While trigonal planar coordination is an unusual exception in the polyhalide chemistry,^1^ there are a few known precedents. One example is the free planar [F(ClF_3_)_3_]^−^ anion. In the crystal structure of the Cs salt it shows, however, a distorted *C*_3_-symmetry with a dihedral angle of 136.4(2)°.^17^ Other examples are the poly(hydrogenhalide)halogenates of the general type [X(HY)_3_]^−^ (X, Y = F, Cl, Br, I). For the [F(HF)_3_]^−^ anion (X, Y = F) a planar structure was predicted to be slightly more stable, but only the K^+^-salt shows a planar anion, whereas several other solid-state structures show pyramidal anion structures.^23^ A relaxed surface scan for [X(HF)_3_]^−^ (X = F, Cl) revealed trends similar to those for the polyhalide species (Fig. S6, ESI†). With X = F, the planar structure is more stable than a pyramidal structure, but with X = Cl, the pyramidal structure is slightly favored (MP2/def2-TZVPP: 0.5 kJ mol^−1^). Note, very recently a quantum-chemical investigation has been published which predicts a planar tetracoordinated structure of a fluorine atom in *e.g.* [FIn_4_]^+^ or [FTl_4_]^+^.^24^

A natural bond orbital (NBO) analysis for the planar [F(ClF)_3_]^−^ revealed a pure 2p-type lone pair on the central fluoride ion perpendicular to the molecular plane, while the two other 2p-orbitals (82 kJ mol^−1^) as well as the 2s-orbital (26 kJ mol^−1^) show correlation with the σ*(Cl–F) orbitals of the ligands (Fig. S7, ESI†). For the chloride centered molecules symmetry allowed sp-hybridization is observed at the central ion in addition to overall stronger correlation effects between the central ion and the ligands ([Cl(ClF)_3_]^−^ 134 kJ mol^−1^ per ligand), ([F(ClF)_3_]^−^ 107 kJ mol^−1^ per ligand). If [Cl(ClF)_3_]^−^ is forced into a planar geometry the correlation energy per ligand drops by 8 kJ mol^−1^. This observation is consistent with a general trend in main group chemistry that elements of the second period have larger s-valence orbital contributions to their bonds and larger bond angles than the higher homologues.^25^

The trigonal planar structure of the fluoridohalogenates is also consistent with their high ionic bond character, as shown by an analysis according to the atoms in molecules (AIM) scheme and the electron localization function (ELF) for the [F(ClF)_3_]^−^ anion.

2D-maps of the ELF in a plane containing one F–Cl–F unit (Fig. 4) were obtained from periodic solid-state calculations (for details see the ESI†). The valence shell of the central fluoride ion (left in Fig. 4) appears almost symmetrical as expected for a non-covalently bound atom. In contrast, the fluorine atom of the ClF unit (right in Fig. 4), as well as the chlorine atom, shows clear signs of lone pairs. Finally, the different ELF values at the BCPs – 0.6 for the short contact and 0.2 for the long one – confirm that a covalently bound ClF molecule (with large charge-shift contribution) is electrostatically bond to one fluoride ion.^26^

**Fig. 4:**
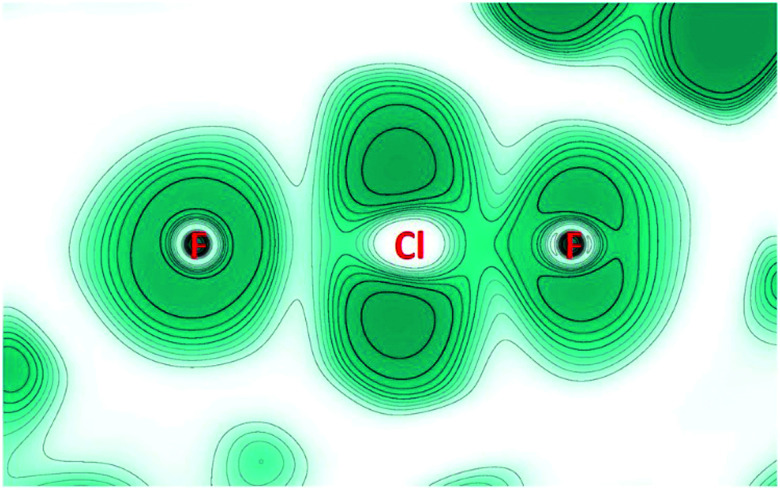
Electron localization function (ELF) calculated for a plane containing a F–Cl–F unit of the [F(ClF)_3_]^−^ anion. Color is ranging from white (0.0) to blue (1.0). Contours are drawn for values from 0.1 to 1.0 in intervals of 0.1. The fluorine atom to the left is the central atom of the anion. For the chlorine atom, no inner shell is seen due to the use of a pseudopotential.

A topological AIM analysis indicates that the bond in a ClF ligand has a strong charge-shift character due to the repulsion between electrons in the lone pairs and the σ-bond. This is best depicted by the ratio of the potential and kinetic charge density (|*V*|/*G*) at the bond critical point (BCP), which is between 1.6 and 1.7 and thus right in the range between ionic (<1.0) and covalent (>2.0) interactions. For the longer Cl–F contacts in [F(ClF)_3_]^−^, the electron density at the BCPs (ρBCP) are significantly lower, indicating a non-shared interaction. The |*V*|/*G* is about 1.0, also suggesting that this interaction is mainly of ionic character.

In conclusion, we report on the first non-classical Cl(i) fluoridochlorate. Additionally, we synthesized the corresponding heptachloride anion. Analysis of the electronic structure and bonding situation revealed an unusual planar minimum structure of the [F(ClF)_3_]^−^ anion. More in-depth quantum-chemical analysis shows the geometry dependence on the central halide ion which can be called fluorine specific.

**Caution!** Chlorine monofluoride is extraordinarily reactive and can react violently with organic materials under the formation of HF. Similarly, [NMe_4_][F(ClF)_3_] can decompose violently under certain conditions when exposed to organic materials. Exposure to acidic compounds (*e.g.* water or boron trifluoride) greatly enhances the reactivity.

We gratefully acknowledge the ZEDAT at Freie Universität Berlin for providing computing resources. Additionally, we are grateful for donations of chemicals from the Solvay Company. PP acknowledges VCI for providing PhD funding (Kekulé Fellowship). Funded by Deutsche Forschungsgemeinschaft (DFG, German Research Foundation) Project-ID 387284271 – SFB 1349. We also gratefully acknowledge support of the ERC-CoG project “HighPotOx” – Project-ID 818862.

## Conflicts of interest

There are no conflicts to declare.

## Supplementary Material

CC-057-D1CC01088C-s001

CC-057-D1CC01088C-s002
