# Supplementary material for: Non-classical polyinterhalides of chlorine monofluoride: experimental and theoretical characterization of [F(ClF)3]−
Source: Chem Commun (Camb). 2021 Apr 7;57(39):4843–6. doi: 10.1039/d1cc01088c (PMC8117328; doi:10.1039/d1cc01088c)
Supplement: CC-057-D1CC01088C-s001 [file CC-057-D1CC01088C-s001.pdf]

Electronic Supplementary Material (ESI) for ChemComm.
This journal is © The Royal Society of Chemistry 2021

Supporting Information

Non-classical polyinterhalides of chlorine monofluoride: Experimental and theoretical characterization of $[F(ClF)_3]^-$

Patrick Pröhm,[a] Nico Schwarze,[a] Carsten Müller,[a] Simon Steinhauer,[a] Helmut Beckers,[a] Susanne M. Rupf,[a] and Sebastian Riedel*[a]

We present the synthesis and characterization of the first non-classical Cl(I) polyinterhalide $[NMe_4][F(ClF)_3]$ as well as the homologous polychloride $[NPr_3Me][Cl_7]$. Both salts were obtained from the reaction of the corresponding ammonium chlorides with ClF or Cl_2, respectively. Quantum-chemical investigations predict an unexpected planar structure for the $[F(ClF)_3]^-$ anion.

Contents

General Information 2
Experimental Section 3
Crystallographic Section 5
Computational Section 7
References 14

Caution!

Chlorine monofluoride is extraordinarily reactive and can react violently with organic materials under the formation of HF. Similarly, $[NMe_4][Cl_3F_4]$ can decompose violently under certain conditions when exposed to organic materials. Exposure to acidic compounds (e.g. water or boron trifluoride) greatly enhances the reactivity.

General Information

All experiments were performed under rigorous exclusion of moisture and oxygen using standard Schlenk techniques. Solids were handled in a dry box under argon atmosphere (O_2 < 0.5 ppm, H_2O < 0.5 ppm). ClF experiments were performend in 8 mm PFA tubes with a stainless steel vacuum line, previously passivated with F_2. Propionitrile was dried over Sicapent® prior to use. $[NMe_4]Cl$, and $[NPr_3Me]Cl$ were dried overnight at 120 °C under dynamic vacuum. All other chemicals were used as purchased. ClF was synthesized as described elsewhere.[1]

Raman spectra were recorded on a Bruker MultiRAM II equipped with a low-temperature Ge detector (1064 nm, 30-80 mW, resolution, 4 cm^{-1}). Spectra of single crystals were recorded at −196 °C using the Bruker RamanScope III. Crystal data were collected on a Bruker D8 Venture diffractometer with a Photon 100 CMOS area detector with MoK_α radiation. Single crystals were picked at –80 °C under nitrogen atmosphere and mounted on a 0.15 mm Mitegen micromount using perfluoroether oil diluted with perfluorohexane. The structures were solved with the ShelXT[2] structure solution program using intrinsic phasing and refined with the ShelXL[3] refinement package using least squares minimizations by using OLEX2.[4] For visualization the Diamond V3.0 program was used.[5]

For -structure optimizations the program packages Turbomole V7.3,[6] Gaussian G16[7] and Molpro2019[8] were used. Thermochemical data on second-order Møller-Plesset perturbation theory (MP2) with spin-component scaling (SCS)[9] and on DFT level with the hybrid functional B3[10]LYP[11] and dispersion correction by Grimme (D3)[12] and Becke-Johnson damping (BJ)[13] with the triple-ζ basis set def2-TZVPP[14] were obtained with the Turbomole V7.3. As a validation for minimum structures, harmonic frequencies were calculated as implemented. Relaxed surface scans and NBO analyses[15] were performed with the Gaussian G16 software package with tight convergence criteria. Structure optimizations on CCSD(T) level were done with the Molpro2019 program. For fluorine the triple ζ basis set aug-cc-pVTZ[16] was used. For chlorine an individually parametrized basis set with the following ECP was used:

```
s,Cl,104.3829980,10.9005580,2.2685170,0.9567350,0.3943800,0.1380120,0.0591000;
c,1.3,0.0031560,0.0239720,-0.3310080;
p,Cl,17.9293820,3.2048610,1.5221960,0.6753990,0.2541180,0.0787660,0.0376000;
c,1.3,0.0029790,-0.0600800,0.0690590;
d,Cl,1.0460000,0.3440000,0.1350000;
f,Cl,0.7060000,0.3120000;
```

```
ECP,Cl,10,3;
1;                                  ! f-ul potential
2,1.000000000,0.000000000;
2;                                  ! s-ul potential
2,6.394300000,33.136632000;
2,3.197100000,16.270728000;
2;                                  ! p-ul potential
2,5.620700000,24.416993000;
2,2.810300000,7.683050000;
1;                                  ! d-ul potential
2,5.338100000,-8.587649000;
```

All periodic solid-state calculations as well as molecular calculations were performed with the CRYSTAL17[17] program, using the B3LYP DFT functional. An energy-consistent, multi-electron fit, quasi relativistic Stuttgart–Cologne pseudo potential with a chemically inactive [Ne] core was utilized for chlorine.[18] The valence electrons of chlorine were represented by a triple-ζ basis set, (6s5p2d)/[4s3p2d], that was applied successfully for polychloride systems before[19] as well as for solid chlorine[20] and was derived from a (6s6p)/[3s3p] basis set by Dolg[21] (for details about this basis set and its derivation, see Ref.[20] and the corresponding Supporting Information). For all other atoms Dunning's cc-pVDZ[22] basis set was used. In the periodic calculations, the first Brillouin zone was sampled using an 6×6×6 Monkhorst-Pack grid. Coulomb and exchange integral thresholds were set to 8, 8, 8, 8, and 16 using the TOLINTEG keyword. QTAIM Analysis was performed

with the TOPOND code developed by Gatti[23] and recently implemented in CRYSTAL17; data for 2D-maps of the ELF were calculated with the CRYSTAL17 program and visualized with Gri.[24]

Experimental Section

Synthesis of $[NMe_4][Cl_3F_4]$

Tetramethylammonium (20 mg, 0.18 mmol, 1 eq) was dissolved in dichlorofluoromethane (0.15 ml) in a 20 cm long 8 mm PFA reactor. Chlorine monofluoride (5 eq) was added at −196 °C and the reactor was flame-sealed. Then it was allowed to warm to −20 °C until the solid was fully dissolved and subsequently slowly cooled to −80 °C. An ethanol filled Dewar was used to slow the cooling rate. Single crystals were obtained after 2 weeks.

Raman: $\tilde{\nu}$ [cm^{-1}] = 3043 (m), 2960 (m), 2328(s), 950 (m), 675 (s), 641 (s), 615 (s).

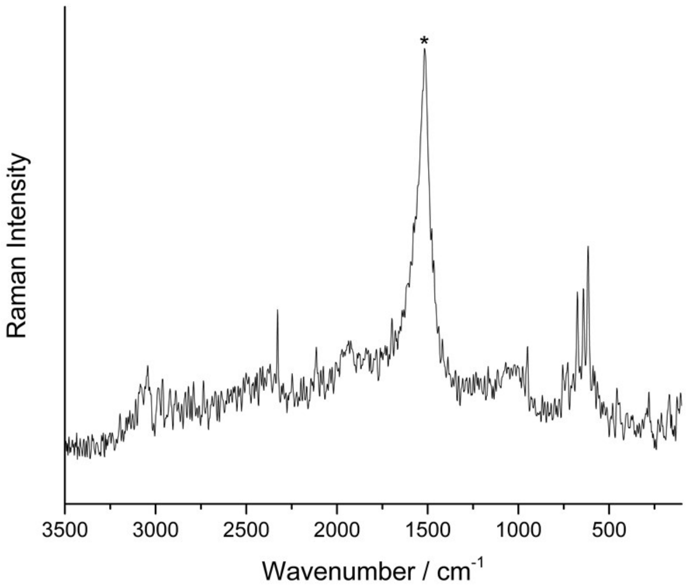

Figure S1 Raman spectrum (77 K) of $[NMe_4][Cl_3F_4]$. Band highlighted with asterisk corresponds to liquid oxygen from the measurement setup.

Synthesis of [NPr_3Me][Cl_7]

Tripropylmethylammonium chloride (660 mg, 3.42 mmol, 1 eq) was dissolved in an excess of chlorine (1.23 g, 17.6 mmol, 5.1 eq) by condensing Cl_2 onto the solid and subsequently allowing it to warm to r.t. For the growth of single crystals, the sample was slowly cooled to −40 °C. An ethanol filled Dewar was used to slow the cooling rate. Single crystals were obtained after several days.

Raman: $\tilde{\nu}$ [cm^{-1}] = 2989 (w), 2948 (w), 2935 (w), 1458 (w), 467 (vs), 444 (vs), 151 (w).

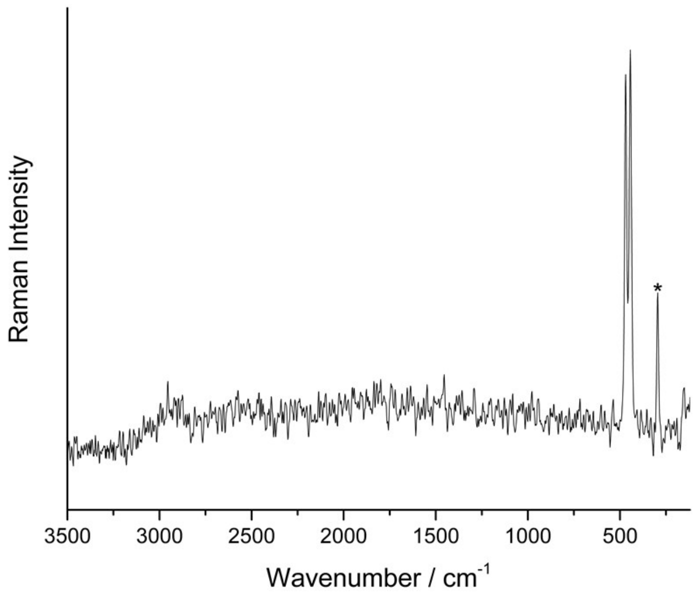

Figure S2 Raman spectrum (77 K) of [NPr_3Me][Cl_7]. Band highlighted with asterisk corresponds to decomposition product [Cl_3]$^-$.

Crystallographic section:

Table S1. Crystallographic details

Empirical formula	$C_{10}H_{24}Cl_7N_7$	$C_4H_{12}Cl_3F_4N$
Formula weight	406.45	256.50
Temperature/K	100.0	100.0
Crystal system	triclinic	orthorhombic
Space group	*P*-1	$Pna2_1$
a/Å	9.2183(6)	11.8381(2)
b/Å	9.6315(6)	10.0385(2)
c/Å	11.5543(8)	9.3211(2)
α/°	67.512(2)	90
β/°	88.551(2)	90
γ/°	89.108(2)	90
Volume/Å^3	947.53(11)	1107.69(4)
Z	2	4
ρ_{calc}g/cm^3	1.425	1.538
μ/mm^{-1}	1.033	7.675
F(000)	420.0	520.0
Crystal size/mm^3	0.3 × 0.22 × 0.15	0.547 × 0.334 × 0.296
Radiation	MoKα (λ = 0.71073)	CuKα (λ = 1.54178)
2Θ range for data collection/°	4.42 to 52.812	11.558 to 144.478
Reflections collected	27183	17449
Independent reflections	3889 [R_{int} = 0.0283, R_{sigma} = 0.0168]	2178 [R_{int} = 0.0491, R_{sigma} = 0.0251]
Data/restraints/parameters	3889/0/167	2178/1/114
Goodness-of-fit on F^2	1.131	1.069
Final R indexes [I>=2σ (I)]	R_1 = 0.0301, wR_2 = 0.0762	R_1 = 0.0199, wR_2 = 0.0517
Final R indexes [all data]	R_1 = 0.0325, wR_2 = 0.0777	R_1 = 0.0204, wR_2 = 0.0523
Largest diff. peak/hole / e Å^{-3}	0.97/-0.85	0.17/-0.15
Flack parameter	-	-
BASF	-	0.495(15)
CCDC deposition numbers	2031712	2060326

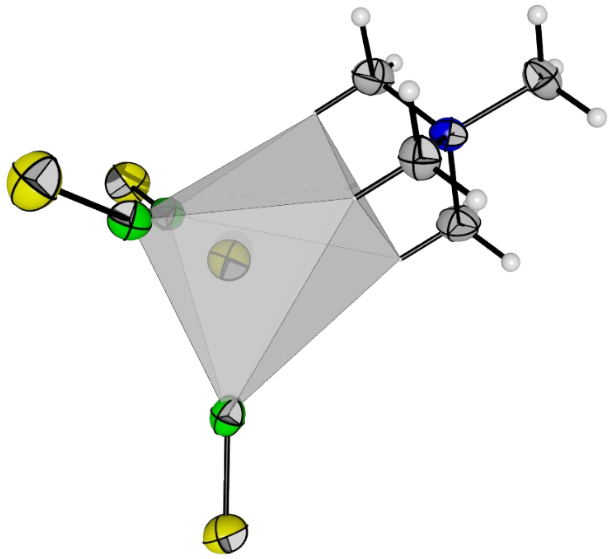

Figure S3. Coordination octahedron of the central fluoride ion in $[NMe_4][Cl_3F_4]$ with three ClF ligands and three hydrogen bonds.

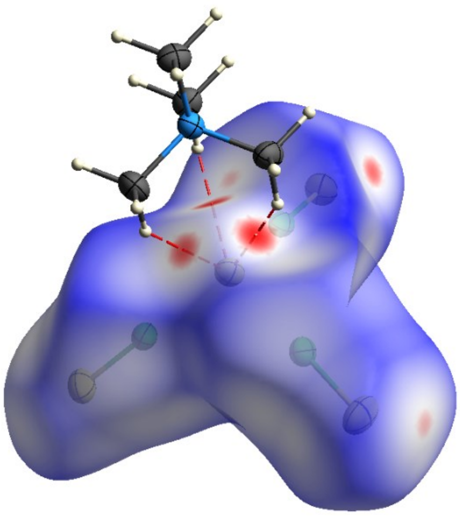

Figure S4. Hirshfeld surface of $[NMe_4][[Cl_3F_4]$, showing three close cation anion contacts via hydrogen bonding.

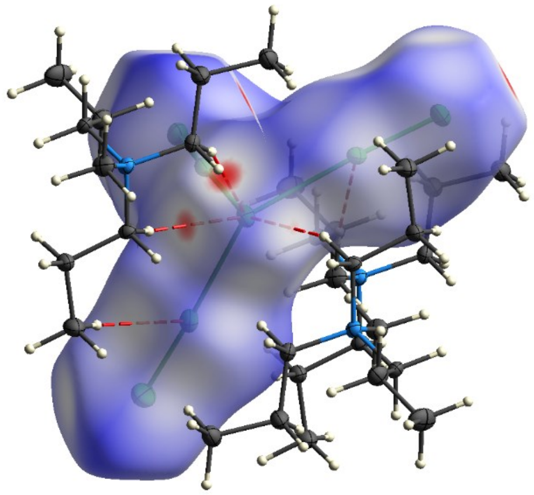

Figure S5. Hirshfeld surface of $[NPr_3Me][[Cl_7]$, showing five close cation anion contacts via hydrogen bonding.

Computational Section

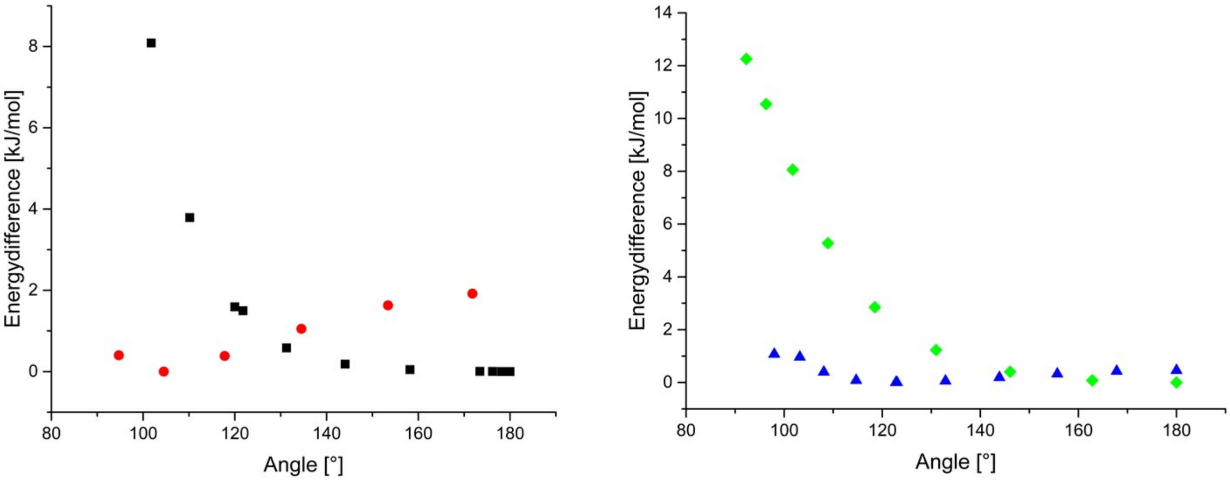

Figure S6. Relaxed surface scans (MP2/def2-TZVPP) of $[F(ClF)_3]^-$ (left, black squares), $[Cl(Cl_2)_3]^-$ (left, red circles), $[F(HF)_3]^-$ (right, green diamonds) and $[Cl(HF)_3]^-$ (right, blue triangles) depending on the dihedral angle.

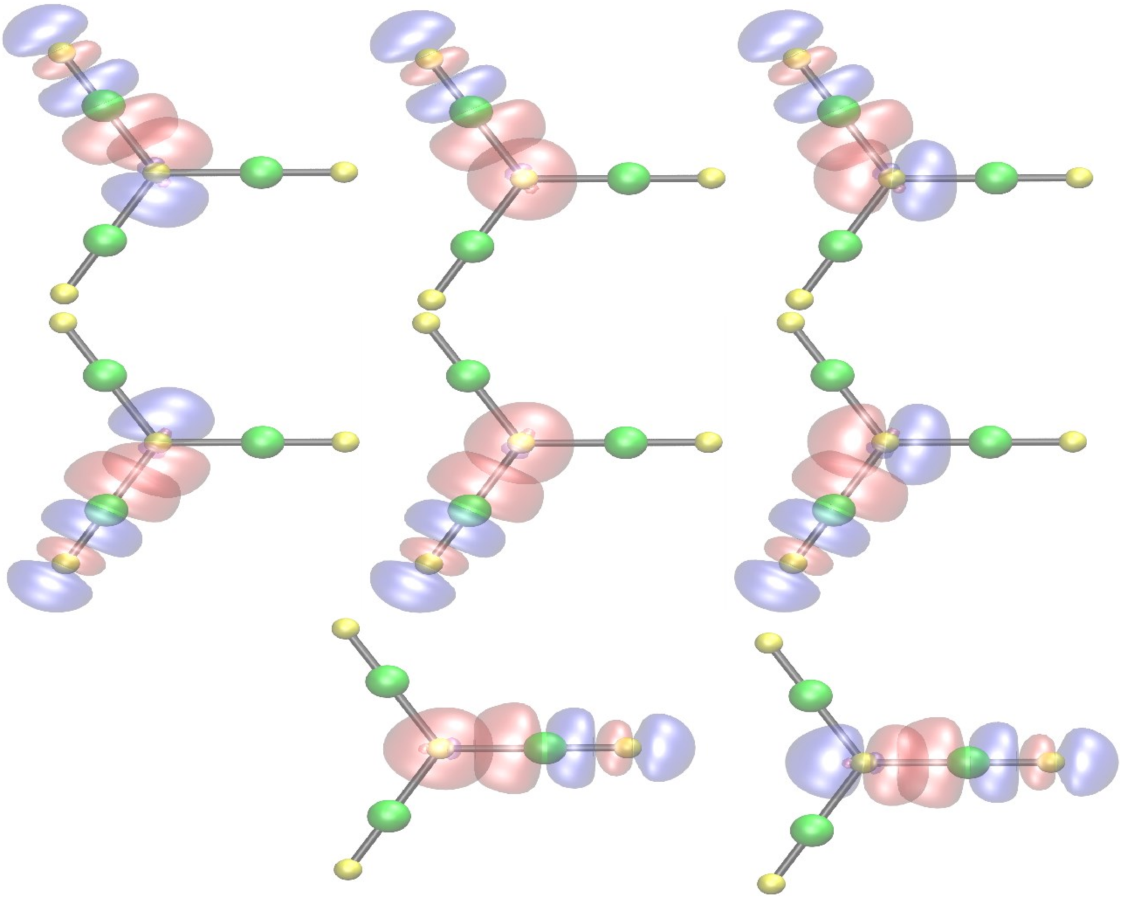

Figure S7. NBOs showing the bonding interactions between the central fluoride ion and the ClF ligands.

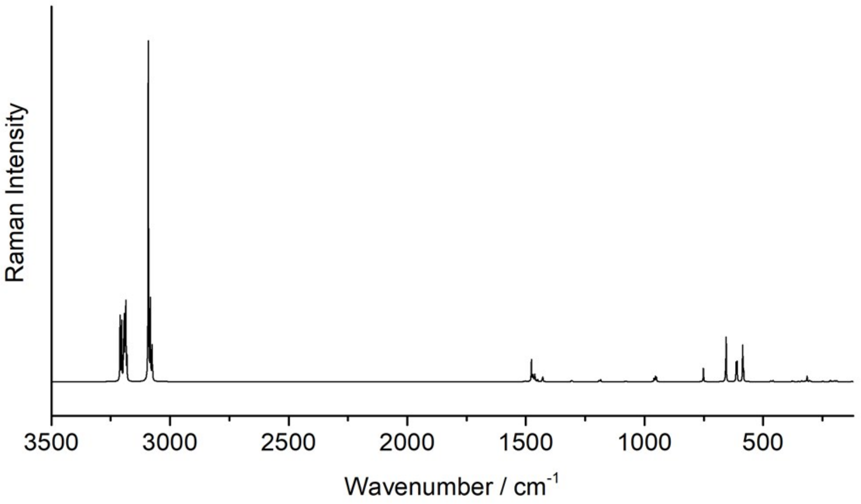

Figure S8 Calculated Raman spectrum of $[NMe_4][F(ClF)_3]$ (periodic solid-state calculation B3LYP-D2).

Frequency [cm⁻¹]	Raman Intensity [arb. u.]	Description
54.18	0.06	
61.37	2.82	
66.68	4.53	
73.62	0.79	
85.79	0.20	
86.88	---	
89.23	1.17	
91.00	0.64	
91.27	0.57	
93.98	0.10	
104.38	0.01	
107.30	---	
116.09	0.34	
117.42	0.23	
125.03	0.06	
188.33	1.98	
118.90	---	
189.01	1.28	
192.66	0.98	
193.81	1.54	
199.02	1.89	
212.73	0.63	
212.96	1.12	
216.99	4.05	
217.56	0.02	
246.73	1.47	CH_3-rotation, F-Cl-F-bending
248.88	0.89	
297.90	1.22	
302.08	2.55	
304.05	0.03	
305.62	3.34	
314.42	7.07	
314.55	0.21	
315.11	10.54	
317.35	0.49	CH_3-rotation, F-Cl-stretching
321.81	2.80	
325.53	0.14	
333.73	0.42	
333.86	0.27	
334.63	0.09	
337.30	3.00	
339.03	0.52	
348.02	---	
352.09	0.07	
460.25	0.11	C-N-C bending
463.98	0.49	
467.01	2.51	
467.36	0.17	
582.73	33.49	F-Cl-stretching
583.78	0.24	
586.45	106.13	

609.82	56.86	
611.31	1.02	
613.15	17.63	
613.83	43.89	
656.16	132.40	
658.95	6.01	
659.16	12.91	
663.30	0.75	
751.77	40.83	
949.45	4.53	
950.34	10.49	
950.52	0.54	
951.63	0.24	C-N-stretching
954.45	14.63	
955.33	0.14	
956.21	2.12	
959.44	3.92	
960.58	6.68	
1184.71	5.82	C-H-bending
1191.68	2.56	
1427.62	6.34	
1429.14	5.74	
1429.19	5.54	
1430.93	4.72	
1431.39	0.03	
1431.77	0.34	
1450.07	5.69	
1450.31	0.14	
1450.57	0.17	
1461.82	5.66	
1462.13	1.38	
1462.21	10.78	
1462.47	3.76	
1464.68	8.00	
1465.47	3.13	CH_3-Bending
1468.94	14.73	
1470.25	9.30	
1472.66	9.20	
1475.41	3.37	
1476.82	44.90	
1477.08	20.16	
1486.44	0.96	
1495.49	0.05	
1495.67	1.61	
1500.47	0.03	
1501.38	0.45	
1502.64	1.28	
1504.18	0.07	
1504.89	0.67	
1507.70	0.95	
3073.62	9.35	
3074.00	10.50	
3076.25	96.88	
3076.35	4.38	
3076.87	1.46	
3082.07	5.75	
3082.47	4.59	
3083.33	233.97	
3090.52	5.21	
3091.10	0.26	
3091.92	1000.00	
3181.37	5.19	
3181.45	2.78	
3181.47	4.67	
3181.90	18.77	C-H streching
3181.93	33.14	
3181.95	3.30	
3186.49	178.15	
3186.60	15.41	
3186.63	6.47	
3186.65	17.76	
3189.56	116.07	
3189.70	0.79	
3189.86	19.73	
3189.89	10.73	
3193.25	32.58	
3193.38	17.95	
3193.43	119.82	
3193.47	3.96	

3195.95	49.18	
3196.17	5.65	
3195.22	29.74	
3196.25	11.77	
3203.93	78.12	
3204.02	9.88	
3204.08	84.65	
3210.69	113.60	
3210.69	29.39	
3210.79	55.78	

xyz-coordinates of optimized (SCS-MP2/def2-TZVPP) $[Cl_3F_4]^-$ anion.

F	0.0030253	0.0054886	0.0014566
Cl	-0.3592242	-0.6223461	2.0362951
Cl	1.8007373	-0.6231479	-1.0177774
Cl	-1.4400692	1.2481127	-1.0179147
F	-0.6450901	-1.1177886	3.6423700
F	-2.5791231	2.2289924	-1.8223426
F	3.2197440	-1.1193112	-1.8220871

xyz-coordinates of optimized (B3LYP-D3BJ/def2-TZVPP) $[Cl_3F_4]^-$ anion.

F	-0.1536391	-0.2668807	-0.1115889
Cl	-0.3802044	-0.6581485	2.0014337
Cl	1.7637205	-0.6601486	-1.0267206
Cl	-1.4531163	1.1971881	-1.0259917
F	-0.5675361	-0.9811748	3.6837986
F	-2.4976014	2.3547186	-1.7599470
F	3.2883767	-0.9855541	-1.7609839

xyz-coordinates of optimized (CCSD(T)) $[Cl_3F_4]^-$ anion.

Cl	0.0009904861	2.1531833330	0.1643768064
F	-0.0047317888	3.8610420453	0.2948078648
F	0.0085650425	-0.0005862369	-0.0001350683
Cl	0.0010431428	-0.9344525242	-1.9478674402
F	-0.0047897103	-1.6748187581	-3.4924261114
Cl	0.0010481618	-1.2190292316	1.7834399958
F	-0.0047944861	-2.1850801623	3.1978478107

xyz-coordinates of optimized (SCS-MP2/def2-TZVPP) $[Cl_2F_3]^-$ anion.

F	-0.9339776	0.6604305	-0.0000001
Cl	-1.1316617	-1.3729277	-0.0000002
Cl	0.9171898	1.5245794	0.0000003
F	2.4829014	2.2932242	-0.0000002
F	-1.3344518	-3.1053063	0.0000001

xyz-coordinates of optimized (B3LYP-D3BJ/def2-TZVPP) $[Cl_2F_3]^-$ anion.

F	-0.9379490	0.6632979	-0.0000001
Cl	-1.1361637	-1.3813141	-0.0000013
Cl	0.9235012	1.5316916	0.0000015
F	2.5047300	2.3217379	-0.0000008
F	-1.3541185	-3.1354134	0.0000007

xyz-coordinates of optimized (CCSD(T)) $[Cl_2F_3]^-$ anion.

Cl	0.0000000000	0.1017656146	1.7597803656
F	0.0000000000	-0.7689394000	3.2780644604
F	0.0000000000	1.1580619590	-0.0000008778
Cl	0.0000000000	0.1017754626	-1.7597836751
F	0.0000000000	-0.7689514221	-3.2780574067

xyz-coordinates of optimized (SCS-MP2/def2-TZVPP) $[Cl_6F]^-$ anion.

F	-0.0003182	0.0000011	0.0016983
Cl	-1.1137745	1.9294117	0.0003593
Cl	-1.1137778	-1.9294109	0.0003517
Cl	-2.1487445	-3.7227928	-0.0008831
Cl	-2.1487433	3.7227934	-0.0008684
Cl	2.2273872	-0.0000136	0.0003203
Cl	4.2979711	0.0000110	-0.0009432

xyz-coordinates of optimized (B3LYP-D3BJ/def2-TZVPP) $[Cl_6F]^-$ anion.

F	0.5361256	0.5360421	0.5356411
Cl	-0.1946310	-0.1945864	2.5106608
Cl	2.5110957	-0.1948964	-0.1947234
Cl	-0.1948466	2.5110478	-0.1946218
Cl	4.3675824	-0.8929151	-0.8921457
Cl	-0.8924256	-0.8922506	4.3671269
Cl	-0.8929006	4.3675586	-0.8919379

xyz-coordinates of optimized (CCSD(T)) $[Cl_6F]^-$ anion.

Cl	1.1363609971	0.0004079770	1.9182020100
Cl	2.1988605108	-0.0007146636	3.7121474025
F	0.0003207373	0.0016776927	-0.0000108523
Cl	1.0928094777	0.0004266974	-1.9433573152
Cl	2.1145455680	-0.0007243406	-3.7608277747
Cl	-2.2289554554	0.0004324577	0.0251499828
Cl	-4.3137929736	-0.0007271627	0.0486915100

xyz-coordinates of optimized (B3LYP-D3BJ/def2-TZVPP) $[Cl_4F]^-$ anion.

F	-0.0000000	0.0000001	-1.2096049
Cl	-1.8478920	0.0000000	-0.1916745
Cl	1.8478920	-0.0000002	-0.1916745
Cl	3.7666507	0.0000001	0.7964769
Cl	-3.7666507	-0.0000000	0.7964769

xyz-coordinates of optimized (CCSD(T)) $[Cl_4F]^-$ anion.

Cl	0.0000000000	1.8222671175	0.3342801156
Cl	0.0000000000	3.6777510085	-0.7106616237
F	0.0000000000	-0.0000016207	1.4047356203
Cl	0.0000000000	-1.8222634965	0.3342776012
Cl	0.0000000000	-3.6777537610	-0.7106598486

xyz-coordinates of optimized (SCS-MP2/def2-TZVPP) $[Cl_4F_3]^-$ anion.

Cl	-0.5610671	-0.9720259	-0.3968604
Cl	-0.4565580	-0.7905466	2.1485504
Cl	1.8736258	-0.7906509	-1.1466457
Cl	-1.6213855	1.2272003	-1.1464218
F	-0.3959032	-0.6851468	3.8620393
F	-2.3480901	2.6965036	-1.6601356
F	3.5093781	-0.6853337	-1.6605263

xyz-coordinates of optimized (B3LYP-D3BJ/def2-TZVPP) $[Cl_4F_3]^-$ anion.

Cl	-0.1826938	-0.3205968	-0.1312649
Cl	-0.1243599	-0.2125720	2.3966331
Cl	2.2206795	-0.2140995	-0.9166975
Cl	-1.2936933	1.8142015	-0.9138059
F	-0.1029684	-0.1689759	4.1418649
F	-2.0856681	3.2672041	-1.4702005
F	3.8738851	-0.1724934	-1.4765202

xyz-coordinates of optimized (CCSD(T)) $[Cl_4F_3]^-$ anion.

Cl	-0.6061668304	-1.0498807873	-0.4286255048
Cl	-0.4555742123	-0.7890568463	2.0886221555
Cl	1.8173179784	-0.7890608114	-1.1257134650
Cl	-1.5919781281	1.1793553813	-1.1257072247
F	-0.3592491688	-0.6222299765	3.8063425420
F	-2.2732606998	2.6931074752	-1.6074556285
F	3.4689110610	-0.6222344348	-1.6074629744

xyz-coordinates of optimized (SCS-MP2/def2-TZVPP) $[Cl_3F_2]^-$ anion.

Cl	0.0000000	-0.0000003	-1.5754267
Cl	-1.9645902	0.0000004	-0.1182676
Cl	1.9645902	0.0000004	-0.1182676
F	3.3979807	-0.0000002	0.9059811
F	-3.3979807	-0.0000002	0.9059811

xyz-coordinates of optimized (B3LYP-D3BJ/def2-TZVPP) $[Cl_3F_2]^-$ anion.

Cl	-0.1636318	-0.0000018	-0.1158603
Cl	-0.2006988	0.0000013	2.3316518
Cl	2.1313584	0.0000025	-0.9665130
F	3.7914329	-0.0000013	-1.6460291
F	-0.2881518	-0.0000007	4.1234376

xyz-coordinates of optimized (CCSD(T)) $[Cl_3F_2]^-$ anion.

Cl	-0.0000000000	-0.0000001980	-1.5957441585
Cl	-1.9418740298	0.0000002739	-0.1256930692
Cl	1.9418740298	0.0000002739	-0.1256930693
F	3.3706197564	-0.0000001249	0.9235652985
F	-3.3706197564	-0.0000001249	0.9235652984

xyz-coordinates of optimized (B3LYP-D3BJ/def2-TZVPP) $[Cl_7]^-$ anion.

Cl	-0.5234752	0.9072869	-0.3718812
Cl	-0.4856117	0.8398819	2.2585893
Cl	1.9696101	0.8410976	-1.2114195
Cl	-1.7130635	-1.2849912	-1.2106431
Cl	-0.4926385	0.8526057	4.3837772
Cl	3.9716452	0.8554413	-1.9244998
Cl	-2.7264664	-3.0113221	-1.9239229

xyz-coordinates of optimized (B3LYP-D3BJ/def2-TZVPP) $[Cl_5]^-$ anion.

Cl	-0.2372058	-0.0000048	-0.4214092
Cl	-0.1667735	-0.0000003	2.0979990
Cl	2.1849543	0.0000056	3.0041587
Cl	-0.3772537	0.0000023	-2.6045621
Cl	4.1965717	-0.0000028	3.8637926

xyz-coordinates of optimized (SCS-MP2/def2-TZVPP) ClF.

Cl	0.0000000	0.0000000	-0.8174630
F	0.0000000	0.0000000	0.8174630

xyz-coordinates of optimized (B3LYP-D3BJ/def2-TZVPP) ClF.

Cl	0.0000000	0.0000000	-0.0054183
F	0.0000000	0.0000000	1.6354183

xyz-coordinates of optimized (CCSD(T)) ClF.

Cl	0.0000000000	0.0000000000	-0.8217078447
F	0.0000000000	0.0000000000	0.8217078447

xyz-coordinates of optimized (SCS-MP2/def2-TZVPP) Cl_2.

Cl	0.0000000	0.0000000	-0.9987069
Cl	0.0000000	0.0000000	0.9987069

xyz-coordinates of optimized (B3LYP/def2-TZVPP) Cl_2.

Cl	0.0000000	0.0000000	-1.0069131
Cl	0.0000000	0.0000000	1.0069131

xyz-coordinates of optimized ((CCSD(T)) Cl_2.

Cl	0.0000000000	0.0000000000	-1.0063722385
Cl	0.0000000000	0.0000000000	1.0063722385

References

1 M. Baudler and G. Brauer, *Handbuch der Präparativen Anorganischen Chemie in drei Bänden, Bd1*, Ferdinand Enke, Stuttgart, 3rd edn., 1975–1981.
2 G. M. Sheldrick, *Acta Cryst. A*, 2015, **71**, 3–8.
3 G. M. Sheldrick, *Acta Cryst.*, 2015, **C71**, 3–8.
4 O. V. Dolomanov, L. J. Bourhis, R. J. Gildea, J. A. K. Howard and H. Puschmann, *J. Appl. Cryst.*, 2009, **42**, 339–341.
5 K. Brandenburg, *DIAMOND*, Crystal Impact GbR, Bonn, 2014.
6 TURBOMOLE GmbH, *TURBOMOLE V7.3. a development of University of Karlsruhe and Forschungszentrum Karlsruhe GmbH*, 2018.
7 M. J. Frisch, G. W. Trucks, H. B. Schlegel, G. E. Scuseria, M. A. Robb, J. R. Cheeseman, G. Scalmani, V. Barone, G. A. Petersson, H. Nakatsuji, X. Li, M. Caricato, A. V. Marenich, J. Bloino, B. G. Janesko, R. Gomperts, B. Mennucci, H. P. Hratchian, J. V. Ortiz, A. F. Izmaylov, J. L. Sonnenberg, D. Williams-Young, F. Ding, F. Lipparini, F. Egidi, J. Goings, B. Peng, A. Petrone, T. Henderson, D. Ranasinghe, V. G. Zakrzewski, J. Gao, N. Rega, G. Zheng, W. Liang, M. Hada, M. Ehara, K. Toyota, R. Fukuda, J. Hasegawa, M. Ishida, T. Nakajima, Y. Honda, O. Kitao, H. Nakai, T. Vreven, K. Throssell, J. A. Montgomery, Jr., J. E. Peralta, F. Ogliaro, M. J. Bearpark, J. J. Heyd, E. N. Brothers, K. N. Kudin, V. N. Staroverov, T. A. Keith, R. Kobayashi, J. Normand, K. Raghavachari, A. P. Rendell, J. C. Burant, S. S. Iyengar, J. Tomasi, M. Cossi, J. M. Millam, M. Klene, C. Adamo, R. Cammi, J. W. Ochterski, R. L. Martin, K. Morokuma, O. Farkas, J. B. Foresman, and D. J. Fox, *Gaussian 16*, Gaussian, Inc., Wallingford CT, 2016.
8 H.-J. Werner, P. J. Knowles, G. Knizia, F. R. Manby, M. Schütz, P. Celani, W. Györffy, D. Kats, T. Korona, R. Lindh, A. Mitrushenkov, G. Rauhut, K. R. Shamasundar, T. B. Adler, R. D. Amos, S. J. Bennie, A. Bernhardsson, A. Berning, D. L. Cooper, M. J. O. Deegan, A. J. Dobbyn, F. Eckert, E. Goll, C. Hampel, A. Hesselmann, G. Hetzer, T. Hrenar, G. Jansen, C. Köppl, S. J. R. Lee, Y. Liu, A. W. Lloyd, Q. Ma, R. A. Mata, A. J. May, S. J. McNicholas, W. Meyer, T. F. Miller III, M. E. Mura, A. Nicklass, D. P. O'Neill, P. Palmieri, D. Peng, K. Pflüger, R. Pitzer, M. Reiher, T. Shiozaki, H. Stoll, A. J. Stone, R. Tarroni, T. Thorsteinsson, M. Wang and M. Welborn, *MOLPRO, version 2019.2, a package of ab initio programs*, molpro_address, 2019.
9 A. Hellweg, S. A. Gruen and C. Hättig, *Phys. Chem. Chem. Phys.*, 2008, **10**, 4119–4127.
10 A. D. Becke, *J. Chem. Phys.*, 1993, **98**, 1372–1377.
11 C. Lee, W. Yang and R. G. Parr, *Phys. Rev. B*, 1988, **37**, 785–789.
12 S. Grimme, J. Antony, S. Ehrlich and H. Krieg, *J. Chem. Phys.*, 2010, **132**, 154104.
13 S. Grimme, S. Ehrlich and L. Goerigk, *J. Comput. Chem.*, 2011, **32**, 1456–1465.
14 a) F. Weigend and R. Ahlrichs, *Phys. Chem. Chem. Phys.*, 2005, **7**, 3297–3305; b) F. Weigend, M. Häser, H. Patzelt and R. Ahlrichs, *Chem. Phys. Lett.*, 1998, **294**, 143–152;
15 E. D. Glendening, J. K. Badenhoop, A. E. Reed, J. E. Carpenter, J. A. Bohmann, C. M. Morales, P. Karafiloglou, C. R. Landis and F. Weinhold, *NBO 7.0*, Theoretical Chemistry Institute, University of Wisconsin, Madison, WI, 2018.
16 F. Weigend, A. Köhn and C. Hättig, *J. Chem. Phys.*, 2002, **116**, 3175–3183.
17 R. Dovesi, A. Erba, R. Orlando, C. M. Zicovich-Wilson, B. Civalleri, L. Maschio, M. Rérat, S. Casassa, J. Baima, S. Salustro and B. Kirtman, *WIREs Comput Mol Sci*, 2018, **8**, e1360.
18 A. Bergner, M. Dolg, W. Küchle, H. Stoll and H. Preuß, *Mol. Phys.*, 1993, **80**, 1431–1441.
19 a) R. Brückner, P. Pröhm, A. Wiesner, S. Steinhauer, C. Müller and S. Riedel, *Angew. Chem. Int. Ed.*, 2016, **55**, 10904–10908; b) R. Brückner, H. Haller, S. Steinhauer, C. Müller and S. Riedel, *Angew. Chem. Int. Ed.*, 2015, **54**, 15579–15583;
20 K. G. Steenbergen, N. Gaston, C. Müller and B. Paulus, *J. Chem. Phys.*, 2014, **141**, 124707.
21 M. Dolg, Universität Stutgart, 1989.
22 T. H. Dunning, *J. Chem. Phys.*, 1989, **90**, 1007–1023.
23 C. Gatti, *Acta Crystallogr A Found Crystallogr*, 1996, **52**, C555-C556.
24 Dan E. Kelley, *gri – scientific graphic program*, 2017.
